# Effect of honey in diabetes mellitus: matters arising

**DOI:** 10.1186/2251-6581-13-23

**Published:** 2014-01-29

**Authors:** Omotayo O Erejuwa

**Affiliations:** 1 Department of Pharmacology, School of Medical Sciences, Universiti Sains Malaysia, 16150 Kubang Kerian, Kelantan, Malaysia

**Keywords:** Diabetes mellitus, Glycemic control, Oxidative stress, Honey, Anti-diabetic drugs, Antioxidants, Vitamin C, Complementary and alternative medicine

## Abstract

Diabetes mellitus remains an incurable disorder in spite of intense research. As result of limitations and unmet goals associated with the use of anti-diabetic drugs, an increased number of diabetic populations globally now resort to complementary and alternative medicine (CAM) such as herbs and other natural products. There has been a renewed interest in the use of honey in the treatment of diabetes mellitus, partly due to an increase in the availability of evidence-based data demonstrating its benefits in diabetic rodents and patients. This commentary aims to underscore some of the research implications, issues and questions raised from these studies which show the beneficial effects of honey in the treatment of diabetes mellitus. Some of the issues highlighted in this article include: considering honey is sweet and rich in sugars, how could it be beneficial in the management of diabetes mellitus? Are the observed effects of honey or combined with anti-diabetic drugs exclusive to certain honey such as tualang honey? Could these beneficial effects be reproduced with other honey samples? Anti-diabetic drugs in combination with honey improve glycemic control, enhance antioxidant defenses and reduce oxidative damage. These effects are believed to be mediated partly via antioxidant mechanism of honey. This raises another question. Could similar data be obtained if anti-diabetic drugs are co-administered with other potent antioxidants such as vitamin C or E? As the evidence has revealed, the prospect of managing diabetes mellitus with honey or antioxidants (such as vitamin C or E) as an adjunct to conventional diabetes therapy is vast. However, more well-designed, rigorously conducted randomized controlled studies are necessary to further validate these findings.

## Background

Diabetes mellitus remains an incurable disorder which is associated with poor quality of life, cardiovascular complications, increased mortality and morbidity [1]. The recent statistics shows that the global prevalence of this disorder continues to rise unabated and thus becoming an epidemic [2]. This is of public health concern due to its social and economic burdens. Even though diabetes has no known cause, complex interplay of several factors including genetic, social, and environmental factors is implicated in its etiology [3]. At the moment, the management of this disorder entails increased physical activity, healthy eating or diet and administration of anti-diabetic drugs and/or insulin. However, the currently available anti-diabetic drugs are far from being satisfactory. This may partly be attributed to the fact that diabetes is a disorder with multifactorial and heterogeneous etiologies. Besides, these agents are costly and, in some cases, not readily available. As a result of these limitations and unmet goals, a large percentage of the population are resorting to CAM [4]. This alternative approach to diabetes therapy includes the use of herbal preparations, dietary components or supplements and other natural products such as honey [5]. Honey is a natural substance produced by bees from nectar. In the last few years, there has been an increased interest in the therapeutic uses of honey. This is largely due to an increase in the availability of evidence-based findings demonstrating the health beneficial effects of honey in treating diverse disease conditions including diabetes mellitus. The aim of this commentary is to underscore some of the research implications, issues and questions raised from the studies which demonstrate the beneficial effects of honey in the treatment of diabetes mellitus.

## Beneficial effects of honey in diabetes mellitus and matters arising

Honey has been shown to scavenge reactive oxygen species, ameliorate oxidative stress and reduce hyperglycemia [6,7]. While honey supplementation in diabetic rats ameliorates renal oxidative stress independent of the dose, its hypoglycemic effect is dose-dependent [8]. This is a bit startling as honey is sweet and rich in sugars and it would not have been expected to exert a dose-dependent hypoglycemic effect. To explain this surprising finding, it is hypothesized that the fructose and oligosaccharides present in honey might in some way contribute to the observed hypoglycemic effect [9,10]. In addition to its effects on oxidative stress and hyperglycemia, honey supplementation ameliorates several metabolic derangements commonly observed in diabetes. These include reduced levels of hepatic transaminases, triglycerides and glycosylated hemoglobin (HbA1c) as well as increased HDL cholesterol [11-13].

A study, the first of its kind, reported the beneficial effects of combining anti-diabetic drugs with honey in diabetes mellitus. Honey administration was found to increase serum levels of insulin while it reduced serum concentrations of glucose and fructosamine in diabetic rats [14]. Even though glibenclamide or metformin reduced hyperglycemia, the administration of these drugs in combination with honey resulted in much lower glycemic levels. On the other hand, unlike honey, these anti-diabetic drugs produced no effect on serum fructosamine concentrations. However, when each of these drugs in combination with honey was administered, there was a significant reduction in serum fructosamine, creatinine, bilirubin, triglycerides and very low-density lipoprotein (VLDL) cholesterol in the diabetic rats. These effects were not observed when glibenclamide or metformin was administered alone [14]. Furthermore, the combination of anti-diabetic drugs with honey also enhanced antioxidant defenses and reduced oxidative damage in the kidney and pancreas of diabetic rats [15-17]. In brief, though data from *in vivo* studies are still limited, these studies reveal that honey could be used as an adjunct to diabetes therapy to achieve better glycemic control, improve metabolic derangements and mitigate oxidative stress-linked diabetic complications.

The maintenance of optimal glycemic control remains the main goal of diabetes management. In spite of this, it is well known that optimal glycemic goal is difficult to achieve as it necessitates the use of multiple anti-diabetic drugs [18]. Even then glycemic control deteriorates in these diabetic patients with time [19]. The limitations of this treatment approach have also been revealed in recent studies showing that diabetic patients who were intensively treated for hyperglycemia had higher episodes of weight gain, hypoglycemia and mortality [20,21]. Available evidence implicates the role of oxidative stress in the etiology of β-cell dysfunction leading to the inability of pancreatic β-cells to secrete sufficient insulin to satisfactorily recompense for insulin resistance [22]. Therefore, these research findings demonstrating that honey as an adjunct to anti-diabetic drugs improves glycemic control and metabolic derangements as well as ameliorates cellular oxidative stress would be of great interest in the management of diabetes mellitus.

However, these studies raise a number of interesting questions and debatable issues. These include: are the observed effects of honey exclusive to a particular honey (such as tualang honey)? Could these findings be generalized to other honey samples that originated from other parts of the globe? To be able to adequately address those questions, it would be vital to have data from studies that compare the effects of various honey of diverse origin in diabetes mellitus. The current literature lacks such studies. Nevertheless, in the absence of such data, the few but limited available data hint that these beneficial effects in ameliorating impaired metabolism are not restricted to tualang honey. Several honey samples from other parts of the world have been shown to reduce hyperglycemia and ameliorate metabolic abnormalities in diabetic rats [23], type 1 and type 2 diabetic patients [24-26]. It is noteworthy to state that majority of these studies investigated the acute effects of honey on hyperglycemia and metabolic derangements. This is very important because a number of studies have shown that honey considerably reduced postprandial hyperglycemia but data demonstrating the marked effect of honey on overall glycemia (measured by HbA1c) are limited.

This leads to another question which is: are there studies that reported the beneficial effect of long-term honey administration in diabetes? This is essential because diabetes mellitus is a chronic disorder. A closer look at the literature reveals that such studies are limited. The only study which can be considered long-term lasted 12 weeks. In the recently published article, Abdulrhman and colleagues reported that honey administration improved glycemia, lipid profile and adiposity in type 1 diabetic patients [27]. The longest duration of study in type 2 diabetes mellitus was 8 weeks. In the study, Bahrami et al. reported the beneficial metabolic effects of honey in type 2 diabetic patients [28]. While the type 1 diabetes study did not measure the HbA1c levels [27], Bahrami and colleagues found that honey supplementation resulted in increased plasma levels of HbA1c in type 2 diabetic patients [28]. This is the only study that reported the potential detrimental effect of honey administration on glycemic control (HbA1c) in diabetic patients. The observed elevated levels of HbA1c following honey supplementation might be due to a number of factors including high doses of honey and unusually low fructose:glucose ratio of the honey which can enhance glycation [29]. In view of the fact that this is the only published data on the long-term effect of honey in type 2 diabetic patients, other similar studies or studies of longer duration are urgently warranted to verify if honey deteriorates glycemic control in type 2 diabetic patients. Similarly, since there are marked differences between type 1 and type 2 diabetes mellitus, additional studies are also needed to validate the effects of honey in type 1 diabetes mellitus.

Honey was shown to complement the antidiabetic and antioxidant effect of standard or conventional anti-diabetic drugs (metformin or glibenclamide). Another question raised by these findings is: are the observed beneficial effects of honey in combination with these drugs exclusive to this particular honey (tualang honey)? Following the publication of those findings, data from another group of researchers reveal that the complementary effect of tualang honey could be reproduced with other honey sample (Ilam honey). In a recent study, metformin combined with Ilam honey markedly produced lower levels of hyperglycemia, bilirubin, triglycerides, total cholesterol, VLDL and LDL and increased high density lipoprotein (HDL) cholesterol. On the other hand, metformin alone neither reduced bilirubin and triglycerides nor increased HDL in diabetic rats [30]. These data obtained with Ilam honey are similar to those obtained with tualang honey and thus suggest administration of other honey samples in combination with anti-diabetic drugs could replicate similar effects. However, there might still be some differences in pharmacological effects due to variations among honey samples [29].

As reported, anti-diabetic drugs in combination with honey improved glycemic control, enhanced antioxidant defenses and reduced oxidative damage [14-17,30]. These effects are believed to be mediated partly via antioxidant effect of honey. This therefore raises another important question: could similar data be reproduced if anti-diabetic drugs are co-administered with other potent antioxidants such as vitamin C or E? At the moment, this remains unclear due to lack of data. However, since the publication of the data showing the beneficial effects of honey in combination with anti-diabetic agents, few clinical studies have investigated the effects of metformin or glibenclamide in combination with vitamin C. Similar to the tualang honey study [14], administration of metformin significantly reduced hyperglycemia but did not reduce HbA1c. In contrast, combination of metformin and vitamin C resulted in considerably much lower glycemic levels and significantly reduced HbA1c levels in diabetic patients [31]. Similarly, compared to the previous studies in which metformin or glibenclamide in combination with honey enhanced antioxidant defenses [15-17], metformin in combination with vitamin C also markedly increased plasma ascorbic acid while no such effect was observed with metformin alone [31]. Another group of independent researchers also reported that administering vitamin C as an adjunct to anti-diabetic drugs markedly improved glycemic control (lower levels of HbA1c), enhanced antioxidant defenses (increased serum levels of ascorbic acid and superoxide dismutase) and reduced oxidative damage (decreased serum concentrations of thiobarbituric acid reactive substances) [32]. The results of these studies suggest that vitamin C, like honey, might be beneficial as an adjunct to anti-diabetic drugs in the treatment of diabetic patients.

Lastly, diabetes mellitus is known to increase the risk of developing hypertension. In view of the effectiveness of honey to enhance antioxidant defenses and protect kidney and pancreas against oxidative damage in diabetic, diabetic-hypertensive and hypertensive rats [7,33,34], honey might serve as a valuable agent (both as a potential anti-diabetic and antioxidant agent) in the management of diabetes mellitus and hypertension [35,36]. Even though previous results of clinical studies that investigated the beneficial effects of antioxidants are inconsistent, as highlighted in this commentary, these potential beneficial effects of honey or vitamin C as an adjunct to conventional diabetes therapy suggest the need to re-examine the concept of oxidative stress in diabetes mellitus [37,38].

## Conclusion

The aforementioned are some of the research implications and issues raised from the studies which show the beneficial effects of honey in the treatment of diabetes mellitus. These research findings reinforce the therapeutic prospects of using honey or other potent antioxidants such as vitamin C or E as an adjunct to standard anti-diabetic drugs in the management of diabetes mellitus. Considering the restrictions associated with the use of antioxidants, other interventions targeted at reducing reactive oxygen species production may also be utilized as an adjunct to conventional diabetes therapy. These honey research findings have raised several questions that will stimulate further research in the future, especially as it relates to the prospect of managing diabetes mellitus via interventions that target both hyperglycemia and oxidative stress. It is worth mentioning that intensive control of hyperglycemia might delay or decrease micro-vascular complications, data demonstrating beneficial effects of tight glycemic control on macro-vascular complications or all-cause mortality are lacking. Therefore, the beneficial effects of honey in diabetes might not only be limited to controlling glycemia but might also extend to improving the associated metabolic derangements in this complicated metabolic disorder. There is no doubt that more well-designed, rigorously conducted randomized controlled studies are necessary to further validate these findings.

## Competing interests

The author declares he has no competing interests.

## Authors’ contribution

OOE wrote the manuscript.
